# Supplementary material for: Effects of Fluoride and Calcium Phosphate-Based Varnishes in Children at High Risk of Tooth Decay: A Randomized Clinical Trial
Source: Int J Environ Res Public Health. 2021 Sep 24;18(19):10049. doi: 10.3390/ijerph181910049 (PMC8508447; doi:10.3390/ijerph181910049)
Supplement: Supplementary file 1 [file ijerph-18-10049-s001.zip › ijerph-1333652-supplementary.pdf]

CLINICAL HISTORY

FILIATION DATA.

- Name and Last name:
- Address (with postal code):
- Phone number:
- Email address:
- Place and date of birth:
- Parents’ name:
- Parents’ job:
- Number of siblings:

ANAMNESIS.

- What happens?
- Since when?
- How does it happen?
- What do you attribute to?

PREVIOUS MEDICAL HISTORY.

- Pregnancy and childbirth: ◯ caesarean section ◯ premature ◯ normal
- Mother’s diseases during pregnancy:
- Treatments the mother underwent during pregnancy:
- Type of breastfeeding: ◯ maternal (duration in months) ____ ◯ artificial ◯ mixed
- Allergic problems:
- Is the child taking any medication?:
- Has the child had surgery for any reason ?:
- Does the child have any systematic disease (e.g. asma, celiac, heart diseases….)?
    - Since when?
    - What treatment do you receive or have you received?:

GENERAL FAMILY HISTORY AND MOUTH.

- Hereditary diseases (Sdre. Down, class III, amelogenesis imperfecta ...):
- Family history of changes in the position of the jaws (Class III, etc.)
- How often do parents go to the dentist?

◯ Annually ◯ Every two years ◯More than two years

- Have parents ever had cavities? (Seals, extractions)

◯ Caries more than two years ago ◯ Caries in the last year ◯ No caries

ORAL HISTORY.

- Has the child already gone to the dentist?: ◯ yes ◯ no ◯ Generalist ◯ Pediatric dentist
- Describe the child's behavior:
- Why dental problems took the child to the dentist?:
- ◯ child uses fluoride toothpaste ……………… ppm ◯ fluoride rinses ◯ fluoride tablets

- Previous treatments.
- Habits:
    - How many times does the child brush his/her teeth each day? ◯ None ◯ One ◯ Two ◯ More than two
    - At what time of the day do you brush your teeth? (several options are valid)

      ◯ After breakfast ◯ After lunch ◯ After dinner ◯Others
    - Food (fill in the diet sheet in Annex 1).
        - Breakfast place ◯ Home ◯ School ◯ Bar / Restaurant ◯Other
        - Food place ◯ Home ◯ School ◯ Bar / Restaurant ◯Other
        - Dinner place ◯ House ◯ School ◯ Bar / Restaurant ◯Others
    - Do you drink bottled water? ◯ yes ◯ no What brand?
    - Has the child used a pacifier, bottle or bites his nails, for how long ?:

PSYCHOLOGICAL ASPECTS.

- Has the child suffered from psychological development disorders?:
- How would you define your child's behavior?: ◯ normal ◯ nervous ◯ consented ◯ capricious ◯ mature ◯ rebellious.

EXTRAORAL EXPLORATION.

- Functional examination: chewing, phonation and swallowing.
- Face scan:
    - Front view: symmetry, facial thirds and lip sealing.
    - Lateral view: profile and lip position.

INTRAORAL EXPLORATION.

- Halitosis (local or general):
- Mucosal exploration:
- Exploration of the gum:
- Exploration of the language:
- Examination of braces:
- Dental examination (changes in number, size, color, shape):
- Exploration of habits (atypical swallowing, digital suction and oral breathing):
- Orthodontic examination:
    - Anteroposterior relation (Angle class):
    - Cross relation:
    - Vertical relationship:
    - Dental anomalies:
    - Crowding:

CARIES RISK ASSESMENT:

- Low:
- Moderate:
- High:
- Extreme:

Initial/base line exam date________________________ Caries recall date_________________________			
	Yes	No	Notes
**1. Caries Risk Indicators — Parent Interview****			
(a) Mother or primary caregiver has had active dental decay in the past 12 months			
(b) Child has recent dental restorations (see 5b below)			
(c) Parent and/or caregiver has low SES (socioeconomic status) and/or low health literacy			
(d) Child has developmental problems			
(e) No dental home/episodic dental care			
**2. Caries Risk Factors (Biological) — Parent Interview****			
(a) Child has frequent (more than three times a day) between-meal snacks of sugars/cooked starch/sugared beverages			
(b) Child has saliva-reducing factors present, including:<br>1. Medications (e.g., some for asthma or hyperactivity)<br>2. Medical (cancer treatment) or genetic factors			
(c) Child continually uses bottle - contains fluids other than water			
(d) Child sleeps with a bottle or nurses on demand			
**3. Protective Factors (Nonbiological) — Parent Interview**			
(a) Mother/caregiver decay-free last three years			
(b) Child has a dental home and regular dental care			
**4. Protective Factors (Biological) — Parent Interview**			
(a) Child lives in a fluoridated community or takes fluoride supplements by slowly dissolving or as chewable tablets			
(b) Child's teeth are cleaned with fluoridated toothpaste (pea-size) daily			
(c) Mother/caregiver chews/sucks xylitol chewing gum/lozenges 2-4x daily			
**5. Caries Risk Indicators/Factors — Clinical Examination of Child****			
(a) Obvious white spots, decalcifications, or obvious decay present on the child's teeth			
(b) Restorations placed in the last two years in/on child's teeth			
(c) Plaque is obvious on the child's teeth and/or gums bleed easily			
(d) Child has dental or orthodontic appliances present, fixed or removable: e.g., braces, space maintainers, obturators			
(e) Risk Factor: Visually inadequate saliva flow - dry mouth			

ICDAS REGISTRATION

Initial/base line exam date________________________ Caries recall date_________________________

Age

UPPER RIGHT																	UPPER LEFT
				55	54	53	52	51	61	62	63	64	65				
	18	17	16	15	14	13	12	11	21	22	23	24	25	26	27	28	
M																	M
O																	O
D																	D
B																	B
L																	L

LOW RIGHT																	LOW LEFT
				85	84	83	82	81	71	72	73	74	75				
	48	47	46	45	44	43	42	41	31	32	33	34	35	36	37		
M																	M
O																	O
D																	D
B																	B
L																	L

SALIVA COLLECTION

1. Collect approximately 3.5 mL of saliva	YES NO
2. Time sampling	___h___min
3. How long does it take to collect the sample?	______min
4. Report presence of any disease or injury in the mouth.	YES Specify: ______________________________<br>NO
5. Brushed his/her teeth before coming	YES Specify: ____________________________<br>NO
6. Has eaten or drank anything?	YES Specify: ____________________________<br>NO

PLAQUE INDEX

1. TRIPLAQUE measurement	YES NO
2. Photos	YES NO

1. Paste check	YES NO
2. Toothbrushing check	YES NO

TREATMENT TRACKING SHEET

Date	Treatment	Pediatric dentist
